# Repurposing Antidiabetic Drugs for Cardiovascular Disease

**DOI:** 10.3389/fphys.2020.568632

**Published:** 2020-09-15

**Authors:** Mario Schubert, Sinah Hansen, Julian Leefmann, Kaomei Guan

**Affiliations:** Institute of Pharmacology and Toxicology, Technische Universität Dresden, Dresden, Germany

**Keywords:** SGLT2 inhibitor, GLP1 receptor agonist, dipeptidyl peptidase-4 inhibitor, metformin, cardiovascular outcome trials, drug repurposing

## Abstract

Metabolic diseases and diabetes represent an increasing global challenge for human health care. As associated with a strongly elevated risk of developing atherosclerosis, kidney failure and death from myocardial infarction or stroke, the treatment of diabetes requires a more effective approach than lowering blood glucose levels. This review summarizes the evidence for the cardioprotective benefits induced by antidiabetic agents, including sodium-glucose cotransporter 2 inhibitor (SGLT2i) and glucagon-like peptide-1 receptor agonist (GLP1-RA), along with sometimes conversely discussed effects of dipeptidyl peptidase-4 inhibitor (DPP4i) and metformin in patients with high cardiovascular risk with or without type 2 diabetes. Moreover, the proposed mechanisms of the different drugs are described based on the results of preclinical studies. Recent cardiovascular outcome trials unexpectedly confirmed a beneficial effect of GLP-1RA and SGLT2i in type 2 diabetes patients with high cardiovascular risk and with standard care, which was independent of glycaemic control. These results triggered a plethora of studies to clarify the underlying mechanisms and the relevance of these effects. Taken together, the available data strongly highlight the potential of repurposing the original antidiabetics GLP1-RA and SGLT2i to improve cardiovascular outcome even in non-diabetic patients with cardiovascular diseases.

## Introduction

With the global health problem of overweight and obesity, the prevalence of type 2 diabetes mellitus (T2D) and cardiovascular disease (CVD) is drastically increasing. Diabetes is a major risk factor for the development of micro- and macrovascular complications, including coronary artery disease (CAD), chronic kidney disease (CKD), blindness and stroke (Cosentino et al., 2020). Each of the three individual cardiovascular risk factors, diabetes, a recent heart attack or stroke, leads to a shortened life expectancy. With a combination of these diseases, life expectancy drops significantly further (Emerging Risk Factors Collaboration et al., 2015). Moreover, the clinical observations over the last decade have emphasized the tight correlation between heart failure (HF) and diabetes, which is revealed by the highly elevated risk (2–5 times) of death from heart disease in diabetic patients and the high prevalence (∼30–40%) of a pre-diabetic or diabetic disease in patients with HF (Cosentino et al., 2020).

HF represents a manifold disease, which is diagnosed based on the ejection fraction (EF), the presence of signs or symptoms such as reduced exercise capability or angina pectoris, structural changes in the heart, or the elevated levels of natriuretic peptides, especially brain natriuretic peptide (BNP)/N-terminal pro-brain natriuretic peptide (NT-proBNP) (Cosentino et al., 2020). In light of these criteria, HF is classified to HF with reduced (HFrEF), moderately reduced (HFmrEF), or preserved EF (HFpEF). So far, the treatment strategies for HF in diabetic patients are comparable to non-diabetic patients, despite the presence of additional risk factors including development of atherosclerosis and CKD as well as increased bodyweight or hyperglycaemia. Recommended as first-line therapy for HFrEF are angiotensin converting enzyme inhibitors (ACEi) or β-blockers (BB) and, if necessary, mineralocorticoid receptor antagonists (MRA). Alternatively, angiotensin receptor blockers (ARB) or valsartan/sacubitril, an ARB-neprilysin inhibitor (ARNI) combination, can be used in case of ACEi intolerance (Cosentino et al., 2020). However, the application of these drugs in diabetic HF may lead to complications. BB reduce all-cause mortality and hospitalization in HFrEF patients with congestive HF after myocardial infarction (MI), but their long-term use in T2D patients with CAD was associated with increased all-cause mortality, compared to non-diabetic individuals (Tsujimoto et al., 2017, 2018). Furthermore, the combination of BB and diuretics to treat hypertension favors the development/new-onset of T2D (Cosentino et al., 2020). ACEi or ARB treatment may have beneficial effects on the prevention of T2D in HFrEF patients and protect against kidney damage in patients with hypertension. The use of ARNI in T2D patients with HFrEF had a more favorable effect, which was shown to reduce the risk of cardiovascular death and hypertensive HF, and was associated with improved insulin sensitivity and efficient reduction of glycated hemoglobin (HbA1c) levels (Seferovic et al., 2017). In contrast to these proven medical approaches for HFrEF, no specific therapies are available for HFpEF, although overall survival of these patients is comparable to HFrEF (Bhatia et al., 2006). The high prevalence of HFpEF in patients with T2D is reflected in the diagnosis of HF in 161 of 581 T2D patients (age ≥ 60) with previously unknown HF. Of these patients, 133 patients (82%) were diagnosed with HFpEF (Boonman-de Winter et al., 2012; Cosentino et al., 2020).

A critical aspect for the development and progression of CVD and HF in diabetes is the lowering of impaired blood glucose levels. In patients with T2D, a HbA1c level outside the target range (≥ 7.0%) was the strongest predictor of stroke and acute MI (Rawshani et al., 2018). Epidemiologic studies have demonstrated that a 1% increase of HbA1c levels leads to a 15–18% increased risk for cardiovascular events in T2D patients (Erqou et al., 2013). Clinical trials were performed to examine intensified glucose-lowering therapy in patients with T2D, but the results revealed either no effect or a tendency to worsen the cardiovascular outcome (Erqou et al., 2013). Until 2008, the requirements for diabetes medication were limited to the effectiveness in lowering HbA1c levels and short-term safety in patients (Harrington et al., 2018). However, the signs of adverse cardiovascular events associated with the use of thiazolidinediones, for example, rosiglitazone, led to the initiation of specifically designed cardiovascular outcome trials (CVOTs) (Home et al., 2009; Harrington et al., 2018). The completed CVOTs have evaluated the cardiovascular safety of dipeptidyl-peptidase 4 inhibitors (DPP4i), glucagon-like peptide-1 receptor agonists (GLP1-RA) and sodium-glucose transporter 2 inhibitors (SGLT2i) in T2D patients at risk for HF. Importantly, these drugs were evaluated in patients already receiving standard care with proven benefit for cardiovascular outcome including statins, ACEi, ARB, BB, and glucose-lowering medication such as metformin (Table 1). The Food and Drug Administration (FDA) pretended a maximal hazard ratio (HR) of 1.3 (upper 95% confidence interval, CI) for T2D medications as the primary outcome of three-point major adverse cardiovascular events (3P-MACE), a composite of cardiovascular death, non-fatal MI, and non-fatal stroke (Home et al., 2009).

**TABLE 1:** Cardiovascular outcomes of randomized multicentre clinical trials in T2D patients.

Study	Patient no/follow up	Patient history	Comparison	Parameter	HR (95% CI)
**Metformin**					
UKPDS34 (UKPDS Group, 1998; Holman et al., 2008)	753/10.7 years	T2D, no HF or MI	Metformin vs. diet	T2D-EP*	0.68 (0.53–0.87)
				All-cause mortality	0.64 (0.45–0.91)
				MI	0.61 (0.41–0.89)
			Intensive therapy^#^ vs. diet	T2D-EP*	0.93 (0.77–1.12)
				All-cause mortality	0.92 (0.71–1.18)
				MI	0.79 (0.60–1.05)
SAVOR TIMI 53 (Bergmark et al., 2019): *Post hoc* analysis	12,156/2.1 years	T2D, CVD HF history (21% metformin vs. 11% non-metformin)	Metformin vs. never taken metformin	All-cause mortality	0.75 (0.59–0.95)
				3P-MACE	0.92 (0.76–1.11)
				CV death	0.68 (0.51–0.91)
				MI	1.23 (0.92–1.65)
	2447 pairs of patients^§^ /2.1 years	T2D, CVD HF history (16% both groups)	Metformin vs. never taken metformin	All-cause mortality	0.73 (0.59–0.91)
				3P-MACE	0.92 (0.78–1.10)
				CV death	0.77 (0.59–0.99)
				MI	1.24 (0.95–1.62)
**GLP1 receptor agonists**					
LEADER (Marso et al., 2016b)	9,340/3.8 years	T2D, CVD (81%) HF history (18%)	Liraglutide vs. placebo	All-cause mortality	0.85 (0.74–0.97)
				3P-MACE	0.87 (0.78–0.97)
				CV death	0.78 (0.66–0.93)
				MI	0.86 (0.73–1.00)
				HHF	0.87 (0.73–1.05)
SUSTAIN-6 (Marso et al., 2016a)	3,297/2 years	T2D, CVD (83%) HF history (24%)	Semaglutide (subcutaneous) vs. placebo	All-cause mortality	1.05 (0.74–1.50)
				3P-MACE	0.74 (0.58–0.95)
				CV death	0.98 (0.65–0.93)
				MI	0.81 (0.57–1.16)
				HHF	1.11 (0.77–1.61)
PIONEER 6 (Husain et al., 2019)	3,183/1.3 years	T2D, CVD (85%) HF history (12%)	Semaglutide (oral) vs. placebo	All-cause mortality	0.51 (0.31–0.84)
				3P-MACE	0.79 (0.57–1.11)
				CV death	0.49 (0.27–0.92)
				MI	1.18 (0.73–1.90)
				HHF	0.86 (0.48–1.44)
Harmony Outcomes (Hernandez et al., 2018)	9,463/1.5 years	T2D, CVD (100%) HF history (20%)	Albiglutide vs. placebo	All-cause mortality	0.95 (0.79–1.16)
				3P-MACE	0.78 (0.68–0.90)
				CV death	0.93 (0.73–1.19)
				MI	0.75 (0.61–0.90)
				HHF	0.71 (0.53–0.94)
REWIND (Gerstein et al., 2019)	9,901/5.4 years	T2D, CVD (31%) HF history (9%)	Dulaglutide vs. placebo	All-cause mortality	0.90 (0.80–1.01)
				3-P MACE	0.88 (0.79–0.99)
				CV death	0.91 (0.78–1.06)
				MI	0.96 (0.79–1.15)
				HHF	0.93 (0.77–1.12)
EXSCEL (Holman et al., 2017)	14,752/3.2 years	T2D, CVD (73%) HF history (16%)	Exenatide vs. placebo	All-cause mortality	0.86 (0.77–0.97)
				3P-MACE	0.91 (0.83–1.00)
				CV death	0.88 (0.76–1.02)
				MI	0.97 (0.85–1.10)
				HHF	0.94 (0.78–1.13)
Elixa (Pfeffer et al., 2015)	6,068/2 years	T2D, CVD (100%) HF history (22%)	Lixisenatide vs. placebo	All-cause mortality	0.94 (0.78–1.13)
				3P-MACE	1.02 (0.89–1.17)
				CV death	0.98 (0.78–1.22)
				MI	1.03 (0.87–1.22)
				HHF	0.96 (0.75–1.23)
**DPP4 inhibitors**					
Carmelina (Rosenstock et al., 2019)	6,979/2.2 years	T2D, CVD (57%)	Linagliptin vs. placebo	All-cause mortality	0.98 (0.84–1.13)
				3P-MACE	1.02 (0.89–1.17)
				CV death	0.96 (0.81–1.14)
				MI	1.12 (0.90–1.40)
				HHF	0.90 (0.74–1.08)
Tecos (Green et al., 2015)	14,671/3.0 years	T2D, CVD (100%)	Sitagliptin vs. placebo	All-cause mortality	1.01 (0.90–1.14)
				3P-MACE	0.99 (0.89–1.11)
				CV death	1.03 (0.89–1.19)
				MI	0.95 (0.81–1.11)
				HHF	1.00 (0.83–1.20)
Savor timi 53 (Scirica et al., 2013)	16,492/2.1 years	T2D, CVD (78%)	Saxagliptin vs. placebo	All-cause mortality	1.11 (0.96–1.27)
				3P-MACE	1.00 (0.89–1.12)
				CV death	1.03 (0.87–1.22)
				MI	0.95 (0.80–1.12)
				HHF	1.27 (1.07–1.51)
Examine (White et al., 2013; Zannad et al., 2015)	5,380/1.5 years	T2D, CVD (100%),	Alogliptin vs. placebo	All-cause mortality	0.80 (0.62–1.03)
		acute coronary event		3P-MACE	0.96 (≤ 1.16)
		within 15-90 days		CV death	0.85 (0.66–1.10)
				MI	1.10 (0.88–1.37)
				HHF	1.19 (0.90–1.58)
**SGLT2 inhibitors**					
Empareg-outcome (Zinman et al., 2015)	7,020/3.1 years	T2D, CVD (100%), HF (10%)	Empagliflozin vs. placebo	All-cause mortality	0.68 (0.57–0.82)
				3P-MACE	0.86 (0.74–0.99)
				CV death	0.62 (0.49–0.77)
				MI	0.87 (0.70–1.09)
				HHF	0.65 (0.50–0.85)
Canvas (Neal et al., 2017)	10,142/3.6 years	T2D, CVD (66%), HF (14%)	Canagliflozin vs. placebo	All-cause mortality	0.87 (0.74–1.01)
				3P-MACE	0.86 (0.75–0.97)
				CV death	0.87 (0.72–1.06)
				MI	0.89 (0.73–1.09)
				HHF	0.67 (0.52–0.87)
Declare-timi 58 (Wiviott et al., 2019)	17,160/4.2 years	T2D, CVD (41%), HF (10%)	Dapagliflozin vs. placebo	All-cause mortality	0.93 (0.82–1.04)
				3P-MACE	0.93 (0.84–1.03)
				CV death	0.98 (0.82–1.17)
				MI	0.89 (0.77–1.01)
				HHF	0.73 (0.61–0.88)
Credence (Perkovic et al., 2019)	4,401/2.6 years	T2D, CKD (GFR 30	Canagliflozin vs.	All-cause mortality	0.83 (0.68–1.02)
		to ≤ 90 mL/min per	placebo	3P-MACE	0.80 (0.67–0.90)
		1.73 m^2^)		CV death	0.78 (0.61–1.00)
				HHF	0.61 (0.47–0.80)

*T2D endpoint (T2D-EP, diabetes related endpoint): sudden death, death from hypo- or hyperglycaemia, fatal or nonfatal myocardial infarction, angina, heart failure, stroke, renal failure, amputation, vitreous hemorrhage, retinopathy requiring photocoagulation, blindness in one eye, or cataract extraction. ^#^Intensive therapy: therapy with chloropropamide, glibenclamide, insulin. ^§^Propensity matched patients (2,447 patients with metformin vs. 2,447 patients never taken metformin). 3P-MACE, 3-point major adverse cardiovascular events; CI, confidence interval; CKD, chronic kidney disease; CV death, cardiovascular death; CVD, cardiovascular disease; HHF, hospitalization for heart failure; HF, heart failure; HR, hazard ratio; MI, myocardial infarction; T2D, type 2 diabetes mellitus.

In this review, we summarize the results from clinical studies evaluating the cardioprotective potential of glucose-lowering drugs including metformin, DPP4i, GLP1-RA, and SGLT2i. While broad evidences confirm the safety of glucose-lowering agents from these classes except saxagliptin, several clinical trials strongly indicate drug-specific, beneficial effects of SGLT2i and GLP1-RA on cardiovascular outcome in T2D patients with high cardiovascular risk. Recently reported benefits in non-diabetic patients with cardiovascular diseases further suggest the repurposing of these drugs to improve cardiovascular outcome in non-diabetic patients (Table 2). These findings are highly relevant for everyday clinical practice, considering the prevalence of CVD in diabetic patients and the need for specific therapies for the majority of patients with HFpEF. The clinical data further point toward different cardioprotective mechanisms of SGLT2i and GLP1-RA but leave many questions unanswered. Here, we discuss different hypotheses and potential mechanisms for cardioprotection based on the results from experimental studies, which provide the evidence for direct drug effects on the heart independent of glucose management and not restricted to patients with T2D.

**TABLE 2:** Cardiovascular outcomes in patients without T2D.

Study	Patient no/follow up	Patient history	Comparison	Parameter	Outcome difference (95% CI)
**Metformin**					
Met-remodel (Mohan et al., 2019)	68/12 months	LVH, CAD with insulin resistance or prediabetes	Metformin (12 months) vs. placebo	LVEF (%)	−0.21 (−4.30–3.88)
				LV mass (g)	−4.4 (−7.4 to −1.4)
				NT-proBNP (pg/mL)	305 (−273 to 884)
Camera (Preiss et al., 2014)	173/1.5 years	CAD	Metformin (1.5 years) vs. placebo	cIMT progression (mm/year)	0.007 (−0.006 to 0.020)
GIPS-III RCT (Lexis et al., 2014; Hartman et al., 2017)	379/2 years	STEMI, primary PCI	Metformin (4 months) vs. placebo	LVEF (%)	−1.71 (−3.73 to 0.31)
				NT-proBNP	No change
				MACE (HR)	1.84 (0.68–4.97)
MetCAB (El Messaoudi et al., 2015)	111/24 h	CABG surgery	Metformin (3 days before surgery) vs. placebo	Troponin I (%)	12.3 (−12.4 to 44.1)
				Arrhythmia	No change
				Days in Intensive Care	No change
				Unit	
**GLP1-RA**					
NCT02001363 (Chen et al., 2015; Huang et al., 2017)	92/3 months	STEMI, T2D: 20% in liraglutide, 16% in control	Liraglutide, 30 min before PCI, total 7 days	LVEF (WMD, %)	4.60 (0.84–8.36)
				MACE* (HR)	0.52 (0.21–1.27)
				Infarct size (% LV)	−6.20 (−9.81 to −2.59)
NCT02001363 (Chen et al., 2016b; Huang et al., 2017)	90/3 months	NSTEMI, T2D: 20% in liraglutide, 28% in control	Liraglutide 7 days prior PCI vs. placebo	LVEF (WMD, %)	5.10 (2.58–7.62)
				MACE* (HR)	0.56 (0.20–1.53)
Kyhl et al. (Kyhl et al., 2016; Huang et al., 2017)	334/5.2 years	STEMI 7–11% diabetes	Exenatide i.v. injection	LVEF (WMD, %)	0.00 (−2.42 to 2.42)
				MACE* (HR)	0.89 (0.61–1.28)
NCT01254123 (Roos et al., 2016; Huang et al., 2017)	91/4 months	STEMI	Exenatide 30 min before PCI, followed by 20μg/day for 3 days	LVEF (WMD, %)	−1.20 (−4.74 to 2.34)
				MACE* (HR)	1.17 (0.17–7.93)
				Infarct size (% LV)	−1.80 (−5.79 to 2.19)
Live (Jorsal et al., 2017)	241 total, 167 w/o T2D/24 weeks	HFrEF LVEF ≤ 45%	Liraglutide (24 weeks) vs. placebo	LVEF (%)	−0.80 (−2.1 to 0.5)
				NT-proBNP (pg/mL)	−140 (−317 to 37)
Fight (Margulies et al., 2016)	300 total, 41% w/o T2D/180 days	HFrEF LVEF ≤ 40%, HHF in last 14 days	Liraglutide (180 days) vs. placebo	CV death (HR)	1.10 (0.57–2.14)
				HHF (HR)	1.30 (0.89–1.88)
				LVEF (%)	−0.1 (−2.3 to 2.1)
**SGLT2 inhibitors**					
DAPA-HF (McMurray et al., 2019)	2,605 w/o T2D of 4,744 total	HFrEF: LVEF ≤ 40%, NYHA class II-IV, NT-proBNP ≥ 600 pg/mL (≥400 pg/mL with prev. HHF)	Dapagliflozin vs. placebo	Prim. outcome^#^ no T2D (HR)	0.73 (0.60–0.88)
				Prim. outcome^#^ total (HR)	0.74 (0.65–0.85)
				CV death (HR)	0.82 (0.69–0.98)
				HHF (HR)	0.70 (0.59–0.83)

*MACE defined as death due to all causes, cardiac death, heart failure, re-myocardial infarction, repeated revascularization, and stroke. ^#^Primary outcome: composite of worsening heart failure (hospitalization or an urgent visit resulting in intravenous therapy for heart failure) or cardiovascular death. CAD, coronary artery disease, including previous myocardial infarction/unstable angina and/or previous revascularization by either percutaneous coronary intervention (PCI) or coronary artery bypass graft (CABG) surgery; CI, confidence interval; cIMT, carotid intima-media thickness; HFrEF, heart failure with reduced ejection fraction; HHF, hospitalization for heart failure; HR, hazard rate; LV, left ventricular; LVEF, left ventricular ejection fraction; LVH, left ventricular hypertrophy; MACE, major adverse cardiovascular events; NSTEMI, non-ST-elevation myocardial infarction; NT-proBNP, N-terminal pro-brain natriuretic peptide; NYHA, New York Heart Association; STEMI, ST-elevation myocardial infarction; WMD, weighted mean difference.

## Metformin in Cardiovascular Disease

Metformin was introduced into the pharmaceutical market in 1995 and belongs to the biguanide class, of which several compounds were retracted from the market because of the severe side effect lactic acidosis (Harrington et al., 2018). The FDA classified HF as a contraindication to metformin therapy up to 2006, which stands against broad evidence from clinical trials (Eurich et al., 2013; Retwinski et al., 2018). Today, a broad evidence has proven the beneficial effect of metformin as a gold standard for the therapy of T2D, due to its good tolerability, weight-lowering effect and low risk of hypoglycaemia (Apovian et al., 2019; Han et al., 2019). Different studies revealed that lactic acidosis is barely occurring with metformin (reviewed in Misbin, 2004).

The antidiabetic mechanism of metformin is dependent on the inhibition of gluconeogenesis and glucose output in the liver (Foretz et al., 2019). Recent studies demonstrated that the increased release of glucagon-like peptide-1 (GLP1) from enterocytes and enteroendocrine cells in the intestine is an important mechanism for the glucose-lowering effect of metformin (Glossmann and Lutz, 2019). An immunometabolism-based beneficial effect of metformin may also contribute to the improved outcome in non-diabetic HF patients (Rena and Lang, 2018). Apart from its effect on diabetes and the heart, metformin treatment was shown to extend the lifespan in mice, highlighting a potential anti-aging effect of the drug (Martin-Montalvo et al., 2013). However, this effect was not found in other species (Glossmann and Lutz, 2019). Further results for a potential anti-aging effect of metformin may be expected from the Targeting Aging with Metformin (TAME) trial, which specifically investigates the effect of metformin on the onset of aging-related diseases (MI, congestive HF, stroke, cancer, dementia, death), however, the trial was not yet listed in ClinicalTrials.gov as of May 2020.

### Cardiovascular Outcome of Metformin in Patients With Diabetes

The UKPDS34 trial represents the most important study for the clinical efficacy of metformin, which enrolled overweight (body mass index, BMI ≥ 25 kg/m^2^) patients with newly diagnosed T2D for conventional diet change and therapy with metformin or other intensive glucose-lowering medications (UKPDS Group, 1998). Patients with a recent MI, HF or angina pectoris were excluded. The trial revealed a 36% reduced rate of all-cause mortality and 39% lower incidence of MI in patients treated with metformin compared to conventional diet change therapy (Table 1). In comparison to other intensive glucose-lowering groups treated with insulin, chlorpropamide or glibenclamide, metformin was superior with respect to diabetes-related endpoints (sudden death, death from hypo- or hyperglycaemia, fatal or non-fatal MI, angina, HF, stroke, renal failure, amputation, vitreous hemorrhage, retinopathy requiring photocoagulation, blindness in one eye, or cataract extraction), all-cause mortality and stroke. In addition, metformin treatment was associated with a lower, but non-significant, risk of MI events compared to other intensive glucose-lowering therapies. The beneficial effects of metformin on diabetes-related endpoints, MI and all-cause death were still present after 10-year follow-up without attempts to maintain the previously assigned therapy (Holman et al., 2008). Notably, no differences in HbA1c were remaining between metformin and other groups after 10-year follow-up. In T2D patients with a history of CAD, treatment with metformin, compared to glipizide, was associated with lowered re-occurrence of major cardiovascular events (MI, stroke, coronary angioplasty, coronary artery bypass graft, cardiovascular death, and death from any cause) (Hong et al., 2013).

The REMOVAL trial, a double-blind, randomized, placebo-controlled study, investigated the effect of metformin on the reduction of insulin requirements and the progression of CAD in T1D patients (Petrie et al., 2017). The maximal carotid intima-media thickness (cIMT, a correlative parameter for atherosclerosis) was significantly reduced in patients treated with metformin, but only trends toward lower mean cIMT progression and insulin requirements were observed. Metformin treatment for 3 years led to the reduction of bodyweight and LDL cholesterol as well as the increase of the estimated glomerular filtration rate (eGFR) in the patients (Petrie et al., 2017). Lowering of HbA1c was initially observed after 3 months, but no differences were remaining 12 months after treatment (Petrie et al., 2017). These findings indicate a use of metformin to improve CVD risk management in both T1D and T2D, but do not support a beneficial effect on glycaemic control in T1D patients.

A limiting aspect for the evaluation of metformin on cardiovascular outcome in T2D patients represents the reproducibility as well as the number and size of specific clinical trials (Boussageon et al., 2016). Critiques of the UKPDS34 trial include the lack of a double-blind design, and no placebo treatment in the control group. In addition, some meta-analyses could not confirm the cardioprotective effect of metformin in T2D patients, although including data from the UKPDS34 trial (Griffin et al., 2017; Harrington et al., 2018). These uncertainties may have contributed to the relatively high proportion of T2D patients without metformin treatment at baseline (up to 34%) in the CVOTs performed for DPP4i, GLP1-RA, and SGLT2i (Table 1). The re-analysis of the CVOT data with respect to metformin would provide randomized, placebo-controlled evidence. In the *post hoc* analysis of the SAVOR TIMI 53 trial (Saxagliptin and Cardiovascular Outcomes in Patients With Type 2 Diabetes Mellitus), patients (*n* = 12,156) with T2D and high cardiovascular risk were classified as ever versus never taking metformin during the trial period. Metformin use was associated with lower rates of all-cause mortality and cardiovascular death but not lower rates of 3P-MACE (Bergmark et al., 2019). In the propensity score-matched analysis (2,447 pairs of patients), similar results were obtained. This observation was most apparent in patients without prior HF or moderate to severe CKD. Supporting evidence for the beneficial effect of metformin for the treatment of T2D is further provided by comprehensive meta-analyses of 25 studies in addition to the SAVOR TIMI 53 covering data from 815,639 patients, showing a reduction of all-cause mortality by 26% (vs. various comparators including sulfonylureas, SU) (Bergmark et al., 2019). In another meta-analysis including 40 studies with total 1,066,408 patients, a reduction of all-cause mortality by 33% (vs. placebo) was reported (Han et al., 2019). Moreover, Han et al. confirmed that metformin reduced the rate of cardiovascular death (19%) and the incidence of cardiovascular events (17%) compared to non-metformin therapy, and lowered the incidence of cardiovascular events (19%) in comparison to SU monotherapy.

### Metformin in Patients Without Diabetes

The already mentioned meta-analysis by Han et al. included the use of metformin in non-diabetic patients, but did not reveal a reduction in cardiovascular events (HR: 0.92; 95% CI: 0.28–3.0; I^2^ 69%) (Han et al., 2019). Evidence for a positive effect of metformin in a non-diabetic population with CAD is provided by the MET-REMODEL trial. This study evaluated the effect of metformin (2,000 mg daily) on left ventricular (LV) hypertrophy (LVH) in pre- or non-diabetic patients (*n* = 68) with insulin resistance and CAD, in addition to standard medication (Mohan et al., 2019). Metformin treatment for 12 months led to the reduction in LV mass, bodyweight, subcutaneous adipose tissue, blood pressure and NT-proBNP levels (Table 2). Importantly, the reduction in LV mass is unlikely caused by the reduction of blood glucose level (Rajagopalan and Rashid, 2019). However, most studies in non-diabetic patients reported that metformin treatment had no or only moderately lowering effects on HbA1c levels (Lexis et al., 2014; Preiss et al., 2014; Griffin et al., 2018; Mohan et al., 2019).

Notably, some studies investigating the effect of metformin on atherosclerosis in non-diabetic patients show different results. The CAMERA study (*n* = 173 patients) examined the effect of metformin (1,700 mg daily) over 18 months in patients with CAD and mean BMI < 30 kg/m^2^ but without diabetes, who were treated with statins (Preiss et al., 2014). Atherosclerosis progression was measured by cIMT, carotid plaque score, and other surrogate markers of CVD and T2D. The trial confirmed the reduction of bodyweight, waist circumference, body fat, level of insulin and tissue plasminogen activator as well as moderately lowered HbA1c for patients treated with metformin, compared to placebo. However, several surrogate markers of cardiovascular disease, including primary outcome cIMT, and carotid score, and secondary outcome cholesterol levels (HDL, non-HDL), triglycerides, C-reactive protein (CRP), and fasting glucose were not affected by metformin. These data are different from those reported in T2D patients, showing reductions in cIMT and total cholesterol levels by metformin in two previous studies (Katakami et al., 2004; Meaney et al., 2008). In the study by Katakami et al. (2004) these changes appear to be independent of the glucose-lowering effect, as fasting glycaemia was comparable between the group treated with metformin and the control group. The effect might be attributed to the absence of statin treatment in the study population (Lexis and van der Horst, 2014).

The GIPS-III trial evaluated the effect of 4-month metformin (1,000 mg daily) treatment on LVEF in patients without diabetes (*n* = 380). Treatment was initiated at the time of hospitalization in patients with ST-elevation MI (STEMI), who underwent primary percutaneous coronary intervention (PCI). Metformin had no influence on the LV function, NT-proBNP levels or MACE during the 4-month study period (Lexis et al., 2014) as well as after 2-year follow-up (Hartman et al., 2017). In the MetCAB trial (*n* = 111 patients), metformin was applied for 3 days before coronary artery bypass surgery in non-diabetic patients (El Messaoudi et al., 2015). The results revealed that short-term metformin pre-treatment, although safe, did not seem to be an effective strategy to reduce periprocedural myocardial injury.

Taken together, these studies underline the efficacy of standard care for non-diabetic patients. Based on the available data, it appears that further metformin medication may induce a relatively small benefit for cardiovascular outcome in non-diabetic patients. Therefore, further evidence is needed to clarify whether metformin has cardiovascular benefit in non-diabetes patients with high cardiovascular risk. The VA-IMPACT (Investigation of Metformin in Pre-Diabetes on Atherosclerotic Cardiovascular OuTcomes, NCT02915198), a randomized, placebo-controlled and double-blind study with a total of 7,868 pre-diabetic patients with established CAD will expand our knowledge. The completion of the study is expected for 2024.

## Glucagon-Like Peptide-1-Mediated Cardioprotection

During the last years, clinical trials provided strong evidence for a cardioprotective effect of GLP1-RA in T2D patients (Table 1). GLP1 is a peptide hormone secreted by the intestine in response to food intake. Through its incretin-like activity, the peptide potentiates insulin secretion while inhibiting glucagon release (Drucker, 2018). GLP1 served as a lead structure for the development of stabilized variants of GLP1-RA to overcome the short plasma half-life of the peptide for therapeutic application (Nauck and Meier, 2019). The effect of GLP1-RA on insulin levels is glucose dependent, which strongly limits the risk of hypoglycaemia (Meloni et al., 2013). In addition, GLP1-RA induce weight loss through the reduction of food intake which is relevant for risk reduction in overweight patients (Drucker, 2018). Liraglutide, a GLP1-RA, has been approved for treatment of T2D in 2009 by the European Medicines Agency (EMA), and in 2010 by the FDA and for the treatment of obesity in 2014 (FDA) and 2015 (EMA) (Iepsen et al., 2015). Liraglutide requires daily injection, whereas prolonged half-life and once-weekly dosing was achieved for newer analogs albiglutide, dulaglutide, and semaglutide. Semaglutide is further available as an orally-available formulation (Jensen et al., 2017). In all GLP1-RA CVOTs, the cardiovascular safety was confirmed, and positive outcomes were observed based on the reduction in either 3P-MACE, cardiovascular mortality, or all-cause mortality, albeit to varying degrees for different GLP1-RA (Table 1).

### Effect of GLP1-RA on Cardiovascular Events in Patients With T2D

Seven CVOTs were performed for the GLP1-RA liraglutide (LEADER; Marso et al., 2016b), semaglutide (SUSTAIN-6; Marso et al., 2016a, PIONEER 6; Husain et al., 2019), albiglutide (Harmony Outcomes; Hernandez et al., 2018), dulaglutide (REWIND; Gerstein et al., 2019), lixisenatide (ELIXA; Pfeffer et al., 2015), and exenatide (EXSCEL; Holman et al., 2017; Table 1). A comprehensive meta-analysis integrating the data from all trials was performed by Kristensen et al. (2019), covering a total number of 56,004 patients. The analysis underlined the positive effect of GLP1-RA on cardiovascular outcome by a 12% reduction in all-cause mortality and cardiovascular death, a reduction in hospitalization due to heart failure (HHF) by 9% as well as a 16% reduction in fatal/non-fatal stroke.

Liraglutide is the first GLP1-RA showing a significant reduction in all-cause mortality (15%) and cardiovascular mortality (22%) in the LEADER trial. The LEADER trial is a randomized, placebo-controlled, double-blind study with 9,340 T2D patients, of which 81% had established CVD. The cardioprotective effect of liraglutide in T2D patients compared to all comparator groups with respect to MACE, acute MI, all-cause mortality, and cardiovascular death was proven in a separate meta-analysis including the data from LEADER and six other studies, but with most patients from the LEADER trial (9,340 of 14,608 total patients) (Duan et al., 2019). Notably, subgroup analysis confirmed a significant reduction in MACE with liraglutide versus placebo, but only a beneficial trend versus other comparators (glibenclamide, rosiglitazone, glimepiride, sitagliptin, total 4,170 patients, HR: 0.58; 95% CI: 0.29–1.16; *P* = 0.122).

A major challenge of most GLP1-RA in clinical practice is their need for subcutaneous application, an issue that has been addressed with the development of oral semaglutide. The placebo-controlled trials of oral semaglutide (PIONEER 6) and subcutaneous semaglutide (SUSTAIN-6) revealed that subcutaneous semaglutide induced a greater reduction of 3P-MACE incidence, whereas the oral form led to remarkably stronger reduction in cardiovascular death (Marso et al., 2016a; Husain et al., 2019; Kristensen et al., 2019). Harmony Outcomes and REWIND investigated the effects of albiglutide and dulaglutide, respectively, and reported the reduction of 3P-MACE incidence consistent with the benefits of liraglutide and subcutaneous semaglutide (Hernandez et al., 2018; Gerstein et al., 2019).

In the EXCSEL trial assessing the cardiovascular outcome of exenatide long-acting release, 14,752 patients (73% of enrolled patients had previous CVD) were followed for a median of 3.2 years. Exenatide treatment was associated with a nominally lower rate of 3P-MACE (HR: 0.91; 95% CI: 0.83–1.00; P = 0.06), cardiovascular death (HR: 0.88; 95% CI: 0.76–1.02; P = 0.096), and all-cause mortality (HR: 0.86; 95% CI: 0.77–0.97; *P* = 0.016) (Holman et al., 2017). A meta-analysis of 16 trials comparing the outcome of exenatide to placebo or other active comparators (different DPP4i, other GLP1-RA or insulin) including the data of EXCSEL (total of 22,003 patients) revealed no significant difference in the rate of cardiovascular events between the groups (Bonora et al., 2019). However, separate analysis of the data excluding the EXCSEL study revealed a non-significant trend toward lower rate of cardiovascular events (HR: 0.80; 95% CI: 0.40–1.63) and all-cause mortality (HR: 0.75; 95% CI: 0.30–1.84).

It is worthy to mention that differences in the results were observed among trials of different GLP1-RA. A stronger cardiovascular benefit was induced by liraglutide, semaglutide, albiglutide and dulaglutide, which are highly homologous with endogenous human GLP1, compared to the structurally distinct exendin-based agonists exenatide and lixisenatide (Table 1; Kristensen et al., 2019). A critical aspect that limits the direct comparability of the data represents also the variation in the study populations as well as drug dosing and kinetics of the different agonists, especially with respect to the short half-time of lixisenatide (Standl, 2019). Moreover, the cardiovascular benefit may be restricted to patients with established CVD, because it could not be shown in a meta-analysis of GLP1-RA trials for patients with multiple risk factors but without established CVD (Zelniker et al., 2019b). Only 73% of patients had established CVD at baseline in the EXCSEL trial whereas 81, 83, and 100% of the population in LEADER, SUSTAIN-6, and Harmony Outcomes, respectively, were in secondary prevention. Based on the slight, non-significant risk reduction of 5% for 3P-MACE in T2D patients at cardiovascular risk without former event, the preventive value of GLP1-RA is discussed for this group of patients (Kristensen et al., 2019). Head-to-head trials comparing the drugs in the same study population directly are required to clarify these differences (Drucker, 2018).

Some results obtained in the recent CVOTs provide the evidence that the reductions in HbA1c, blood pressure and bodyweight alone are not sufficient to explain the cardiovascular effects of GLP1-RA (Drucker, 2018). Especially, whereas no major difference in blood pressure, bodyweight, or renal function between the albiglutide and placebo groups was observed over time in the Harmony Outcomes trial, albiglutide was superior to placebo with respect to 3P-MACE and the risk of atherothrombotic events in T2D patients with high cardiovascular risk (Zweck and Roden, 2019). Hypotheses for the cardioprotective activity of GLP1-RA include an anti-inflammatory pathway, the decrease of blood sugar and lipids as well as prevention of hypertension or reduced atherosclerosis (Reed et al., 2018).

### Cardiovascular Effect of GLP1-RA in Patients Without T2D

Although GLP1-RA are cardioprotective in patients with T2D and high cardiovascular risk, recent studies showed that GLP1 levels were increased in patients with acute MI and were correlated with an adverse outcome and early events (Kahles et al., 2020). Different trials investigated the potential of liraglutide (Chen et al., 2015, 2016a,b) and exenatide (Kyhl et al., 2016; Roos et al., 2016) as a medication in patients presenting with non-ST-elevation MI (NSTEMI) and STEMI (Table 2). A meta-analysis of trials enrolling acute MI patients with PCI (<26% T2D patients) confirmed the reduction in infarct size and improvement in LVEF by treatment with GLP1-RA compared to placebo (Huang et al., 2017). A non-significant trend towards lower rates of MACE (HR: 0.72; 95% CI: 0.58–1.06) was found for GLP1-RA treatment. Interestingly, the improvement of LVEF and the reduction in MACE was consistently observed in patients treated with liraglutide, but absent or much less evident in trials with exenatide. These results point towards a cardioprotective effect of GLP1-RA, especially liraglutide, to improve clinical outcome in patients with acute MI. As seen in the CVOTs, the findings for an application of GLP1-RA in T2D patients with acute MI seem to vary between different agonists.

Two smaller trials testing liraglutide in patients with LVEF < 45% with and without T2D did not reveal a benefit on cardiovascular events, although a reduction of bodyweight and improved glycaemic control was observed (Margulies et al., 2016; Jorsal et al., 2017). Notably, both studies reported an increased rate of serious cardiac adverse events in the group treated with liraglutide. This may be due to increased blood pressure and heart rate reported in different studies after injection of GLP1-RA (Drucker, 2018; Standl, 2019). Based on these findings, the use of GLP1-RA is contraindicated in patients with chronic HF.

## DPP4 Inhibitors (Gliptins) in Cardiovascular Disease

DPP4i represent a class of antidiabetics, which are frequently used in combination with metformin to improve glycaemic control in T2D patients. DPP4 is an abundantly expressed transmembrane-spanning exopeptidase. The antidiabetic activity of DPP4i has been attributed to the role of DPP4 in the cleavage and thus the inactivation of the incretins GLP1 and GIP. However, the physiological consequences of DPP4 inhibition are very complex. DPP4 is involved in the cleavage of a variety of peptides including incretins, cytokines, growth factors and neuropeptides. Thereby, the enzyme affects multiple processes in different tissue, which are involved in sympathetic activation, inflammatory processes and regulation of the immune system (Makrilakis, 2019). In addition, DPP4 also exists in a cleaved, soluble form (sDPP4) (Makrilakis, 2019). The activity of circulating sDPP4 was shown to correlate with poor cardiovascular outcome and reduced LVEF in HF patients and animal models, suggesting a protective effect and an improved cardiovascular outcome of DPP4 inhibition (dos Santos et al., 2013).

### Effect of DPP4i on Cardiovascular Outcome in Patients With T2D

All clinical studies carried out with DPP4i confirmed the cardiovascular safety of the substances sitagliptin (Green et al., 2015), alogliptin (White et al., 2013; Zannad et al., 2015), linagliptin (Rosenstock et al., 2019), and saxagliptin (Scirica et al., 2013), however, the drugs showed only a neutral effect with regard to cardiovascular risk in T2D patients with a history of CVD (Table 1). The results of CARMELINA and TECOS did not show beneficial effects of linagliptin and sitagliptin on cardiovascular outcome for the treatment of T2D patients with increased risk for cardiovascular events (Green et al., 2015; Rosenstock et al., 2019). Conflicting results were observed for the DPP4i saxagliptin. In the SAVOR trial, saxagliptin had also no effect on the 3P-MACE although the treatment improved glycaemic control (lower fasting glucose and HbA1c levels) (Scirica et al., 2013). Notably, the rate of HHF was increased in patients treated with saxagliptin. Adverse events were occurring directly after initiation of the treatment, persisted at 12 months and were most pronounced in patients with impaired kidney function (eGFR < 60 mL/min per1.73 m^2^), prior HF, and elevated baseline levels of NT-proBNP (Scirica et al., 2014). These results suggest the contraindication of saxagliptin for patients with high risk.

The EXAMINE trial investigated the effect of alogliptin in T2D patients after acute coronary disease, MI or unstable angina hospitalization within the previous 15–90 days and reported no effect of alogliptin on 3P-MACE (White et al., 2013). A *post hoc* analysis assessed HHF in the EXAMINE trial, in which about 60% of the patients at baseline had a history of HF before the acute coronary syndrome event. Patients with a history of HF at baseline had higher baseline BNP concentrations and lower eGFR values than patients without a HF history (Zannad et al., 2015). The risk of 3P-MACE and HHF was similar for alogliptin and placebo in the whole cohort (White et al., 2013; Zannad et al., 2015). Subgroup analysis showed that alogliptin did not lead to more new HHF or worse outcome for existing HF in patients with the comorbidity of HF. In those patients without a HF history, a slightly increased risk of cardiovascular death and HHF was observed in the alogliptin group. Further analysis based on the BNP levels revealed that the increased HHF rate by alogliptin was observed in the quartile of patients with highest BNP levels (>173.8 pg/mL), importantly, the rate of cardiovascular death was reduced in these patients, suggesting the influence of possible mortality bias. Based on all available data, alogliptin is not associated with increased risk of HF outcomes in T2D patients with recent acute coronary events.

Seen in the broad context of all clinical trials of linagliptin, alogliptin, and sitagliptin, the increased HHF observed with saxagliptin in the SAVOR TIMI 53 trial is likely a compound-specific, rather than a general class effect (Home, 2019). Conclusions about the class-specific effects of DPP4i should be done carefully, due to the structural variations of different DPP4i and the resulting differences in the selectivity toward DPP8 and DPP9 (Riche and Davis, 2015). These differences may lead to altered adverse effect profiles which must be considered for each chemical entity.

### Application of DPP4i in Patients Without Diabetes

Based on their mechanism of action and the influence of DPP4 inhibition on a variety of different peptide hormones, the class of DPP4i could be expected to affect the metabolism of patients similar to GLP1-RA independently of the presence of diabetes. However, evidence of the effect of DPP4i in non-diabetic patients is rare, and to our best knowledge, no reports have been published on the cardiovascular outcome of DPP4i in patients without diabetes.

Single studies confirmed the reduction of postprandial triglyceride-rich lipoprotein (TRL) apoB48 levels and increased levels of GLP1 in healthy individuals after a single dose of sitagliptin (Xiao et al., 2014). Levels of hepatic apoB100, plasma triglyceride, blood glucose and insulin were not significantly altered. Notably, sitagliptin treatment of T2D patients for 6 weeks led to reduced postprandial plasma levels of apoB100, apoB48, triglyceride, VLDL and glucose (Tremblay et al., 2011). Based on the cleavage of BNP by DPP4, it has been speculated that DPP4i may be beneficial for HF associated with increased pressure load by improving vasodilation and protective cardiac cGMP signaling of BNP (Lambeir et al., 2008). However, in the context of the CVOT results in T2D patients, these expectations were not met as no benefit of DPP4i was observed in addition to standard care (Table 1).

## SGLT2 Inhibitors (Gliflozins) in Cardiovascular Disease

Different SGLT2 inhibitors including canagliflozin, dapagliflozin, empagliflozin, and ertugliflozin are approved for the therapy of T2D and have been recently evaluated for their cardiovascular risk profile in clinical trials (Zinman et al., 2015; Neal et al., 2017; Wiviott et al., 2019). SGLT2i act on the renal proximal tube to block the reabsorption of glucose. By this mechanism, the drugs lead to increased urinary glucose excretion, reduced blood glucose levels, and reduction of plasma volume and sodium load (Verma, 2019; Santos-Ferreira et al., 2020). Unexpectedly, SGLT2i induced a 35–40% risk reduction in cardiovascular death in patients already receiving optimal secondary prevention strategies for heart disease (Table 1). These findings encouraged the re-evaluation for the recommendation of SGLT2i as a first-line treatment for T2D patients with risk for heart disease. Moreover, new trials and experimental studies were initiated to investigate the efficacy of SGLT2i to treat HF in the absence of diabetes (McMurray et al., 2019) and the mechanisms behind the strong cardioprotective effect.

### SGLT2i in Patients With T2D

Different CVOTs including EMPAREG-OUTCOME (Zinman et al., 2015), CANVAS (Neal et al., 2017), and DECLARE-TIMI 58 (Wiviott et al., 2019) have been performed to evaluate the cardiovascular risk of empagliflozin, canagliflozin, and dapagliflozin, respectively, in T2D patients with CVD. In these trials, treatment with SGLT2i was performed on top of standard care therapy and led to a lower rate of all-cause mortality as well as remarkable improvement in the cardiovascular outcome (Table 1).

The meta-analysis of all three trials by Zelniker et al. (2019a) (total of 34,322 patients) confirmed an 11% reduction in 3P-MACE for the overall population and a 14% reduction in patients with atherosclerotic cardiovascular disease (ASCVD). The MACE reduction in ASCVD patients was mainly driven by the lowered incidence of cardiovascular death and MI, but not through the reduction of stroke events. Moreover, a 23% reduced rate of the composite of HHF and cardiovascular death was observed in patients treated with SGLT2i independent of the presence of ASCVD. Interestingly, no effect on 3P-MACE was found for canagliflozin (66% of patients had a history of CVD) and dapagliflozin (41% of patients had a history of ASCVD) whereas a significantly lower rate was observed for empagliflozin in EMPAREG-OUTCOME trial (more than 99% of patients had established CVD). These findings strongly support the high efficacy of SGLT2i in T2D patients with established CVD. The broader entry criteria in DECLARE-TIMI 58 and CANVAS resulted in the inclusion of T2D patients with a history of MI and thus, these trials provide information for the use of SGLT2i as secondary prevention (Table 1). Importantly, the beneficial effect of all three SGLT2i on the composite outcome of HHF and cardiovascular death was present in patients with a history of HF, which highlights the potential of SGLT2i as a secondary prevention therapy for heart disease (Zelniker et al., 2019a). Analysis of the cardioprotective effect of dapagliflozin and canagliflozin in relation to heart function revealed that the lowered HHF rates were consistently present for patients with HFpEF or HFrEF, while the benefit on cardiovascular death and all-cause mortality was restricted to HFrEF patients (Figtree et al., 2019; Kato et al., 2019). In addition to clinical trials, data observed from population-based studies confirmed the reduced rates of HHF and all-cause mortality with the SGLT2i therapy compared to other glucose-lowering drugs (reviewed in Santos-Ferreira et al., 2020).

Importantly, the results from EMPAREG-OUTCOME, DECLARE-TIMI 58, and CANVAS further demonstrate the positive effect of SGLT2i on kidney function. Treatment with SGLT2i was associated with a 45% reduction of the progression of renal disease (composite of worsening renal function, end-stage renal disease, and renal death), independent of the presence of ASCVD (Zelniker et al., 2019b). This benefit was observed in patients over a broad range of basal eGFR (60 to > 90 mL/min per 1.73 m^2^) but was most pronounced in patients with preserved renal function (eGFR ≥ 90 mL/min per 1.73 m^2^). The cardiorenal-protective effect of SGLT2i was further highlighted in the CREDENCE trial to assess the renal outcomes of canagliflozin in T2D patients (total 4,401) with albuminuric CKD and GFR of 30 to < 90 mL/min per 1.73 m^2^ (Perkovic et al., 2019). This trial revealed a 34% risk reduction (HR 0.66; 95% CI: 0.54–0.86) of the renal-specific composite of end-stage kidney disease, a doubling of the creatinine level, or death from renal causes (Perkovic et al., 2019). In addition, the results of CREDENCE confirmed the cardioprotective effect of canagliflozin by observing a 20% risk reduction of 3P-MACE and 39% reduction of HHF (Perkovic et al., 2019).

These results provide evidence for the SGLT2i use in a broader population of T2D patients, irrespective of ASCVD, kidney disease or HF and as a first-line therapy after metformin in most patients with T2D (Verma et al., 2019). Further trials are ongoing to examine the potential of empagliflozin in T2D patients with HFpEF including EMPERIAL-Preserved (NCT03448406) and EMPEROR-Preserved (NCT03057951). Overall, EMPAREG-OUTCOME, CANVAS, CREDENCE, and DECLARE-TIMI 58 trials, as well as several observation studies of population data confirm the significant cardiovascular benefit of T2D patients from the SGLT2i therapy. Moreover, the positive effect on multiple cardiovascular risk factors in addition to glycaemic control, such as improved kidney function, and reduction in bodyweight as well as systolic and diastolic blood pressure, highlight the great potential of SGLT2i in the therapy of T2D patients at high cardiovascular risk.

### SGLT2i in Patients Without T2D

Since glucose levels were comparably managed by standard care in the control and SGLT2i treatment groups in the CVOTs, speculations became evident for a diabetes-independent positive effect. These results encourage investigations for the repurposing of SGLT2i for the treatment of patients with CVD in the absence of diabetes (Petrie, 2019). This issue was addressed in the DAPA-HF trial which enrolled 4,744 patients with HFrEF (LVEF < 40%) already receiving standard care medication including ACEi, ARB, BB, and MRA (McMurray et al., 2019). About half of the patients in the trial had no diabetes. Treatment with dapagliflozin in comparison to placebo led to a 26% risk reduction in the primary outcomes including an unplanned HHF, an intravenous therapy for HF or cardiovascular death. Importantly, this effect was similarly observed for patients with T2D and without diabetes with respective risk reductions of 25 and 27% (McMurray et al., 2019). The data further suggest that dapagliflozin improves the primary outcome in patients taking ARNI at baseline, which is known to be more efficient than RAAS inhibition alone, as shown by reducing the incidence of cardiovascular death and HHF in HF patients. Further trials for empagliflozin were initiated in patients with HFrEF and with or without diabetes, including EMPIRE-HF (NCT03198585) and EMPEROR-Reduced (NCT03057977). Although the data of these trials are required to draw final conclusions, the findings of DAPA-HF indicate a remarkable potential of SGLT2i to improve the efficacy of current HF treatments in non-diabetic patients.

## Implications for the Choice of the Antidiabetic Drugs

As T2D is strongly associated with increased risk for development of atherosclerosis, CKD, and HF, treatment of T2D requires a more effective approach and should not exclusively be glucose lowering. The data observed in recent clinical trials confirm the great potential of the antidiabetic drugs SGLT2i and GLP1-RA in terms of reducing cardiovascular events and preventing the progression of kidney disease.

GLP1-RA cause substantial bodyweight reduction, blood pressure reduction, and a reduction in atherosclerosis and inflammation. Since these are all prevalent in patients with HFpEF or obesity, GLP1-RA could benefit these groups of patients. The greatest cardiovascular risk reduction of GLP1-RA (liraglutide, semaglutide) was observed in obese patients with BMI > 30 kg/m^2^. Some evidences suggest a beneficial effect of the GLP1-RA liraglutide on the clinical outcome in patients with acute MI. Although the data from the LIVE and FIGHT trials of GLP1-RA in HFrEF so far are discouraging, future studies should focus on GLP1-RA in patients with HFpEF. Nephroprotection has been observed in two GLP1-RA (liraglutide and semaglutide) CVOTs, therefore, treatment with GLP1-RA liraglutide and semaglutide is associated with a lower risk of renal endpoints, and should be considered for diabetic patients if eGFR is > 30 mL/min per 1.73 m^2^ (Cosentino et al., 2020).

SGLT2i have been proven to be very useful to reduce cardiovascular risk in T2D patients, beside the reduction of bodyweight and blood pressure. The strong benefits of SGLT2i in preventing HF in patients with T2D have been established in the clinical CVOTs, as discussed above. Especially, results obtained with SGLT2i in patients with established HFrEF but without T2D strongly suggest the repurposing of this class of drugs for HF patients without diabetes. New results from the ongoing trials EMPIRE-HF (NCT03198585) and EMPEROR-Reduced (NCT03057977) for empagliflozin in patients with HFrEF with and without diabetes will provide us a wealth of new evidence. Moreover, positive renal outcomes were observed in the CREDENCE trial for canagliflozin in T2D patients with an eGFR of 30–90 mL/min per 1.73 m^2^. As the results of ongoing trials evaluating SGLT2i in patients with CKD (DAPA-CKD, EMPA-Kidney) are expected with great interest, the correlation indicated by the common incidence of T2D, HF and CKD may hint to the question whether the improvement in kidney function may play a direct role in cardioprotection. If the beneficial effects of SGLT2i in non-diabetic patients can be confirmed, they may become important for the prevention of HF in patients with established CKD (Herrington et al., 2018).

The combined use of SGLT2i and GLP1-RA to further improve the cardiovascular outcome of patients with high cardiovascular risk has been investigated in several studies. Treatment of obese, non-diabetic patients with exenatide and dapagliflozin for 52 weeks reduced bodyweight, total adipose tissue volume, LDL cholesterol, triglycerides, systolic blood pressure and the proportion of patients with pre-diabetes (Lundkvist et al., 2017). In a trial with T2D patients, the influence of liraglutide-empagliflozin combination therapy was compared to monotherapy with liraglutide, empagliflozin or insulin as add-on to metformin (Ikonomidis et al., 2020). All treatments led to reductions in HbA1c, total cholesterol, LDL cholesterol and triglycerides. The combination of empagliflozin and liraglutide was associated with the most favorable effects on myocardial functional markers (global longitudinal and radial strains, myocardial work index) and metabolic parameters (BMI, endothelial glycocalyx thickness, central systolic blood pressure) (Ikonomidis et al., 2020).

The benefit of short-term (12–30 weeks) SGLT2i/GLP1-RA combination therapy in patients with T2D was further confirmed in a meta-analysis (1,913 patients) of seven trials, which revealed the greater reduction in HbA1c, bodyweight and systolic blood pressure compared to GLP1-RA or SGLT2i therapy (Mantsiou et al., 2020). However, conclusions on cardiovascular outcome and mortality are not available so far due to the rare number of cardiovascular events and the duration of the trials. Long-term data (104 weeks) are only available from the DURATION-8 trial, which confirmed the beneficial effect of dapagliflozin/exenatide treatment on HbA1c, bodyweight and systolic blood pressure (Mantsiou et al., 2020). A limitation is the use of different SGLT2i/GLP1-RA combinations in each of these studies, especially with respect to the different benefits of GLP1-RA observed in the CVOTs (Table 1).

These studies underline the potential of the SGLT2i/GLP1-RA combination therapy in patients with high cardiovascular risk. However, whether this is associated with improved cardiovascular outcome in terms of all-cause mortality or incidence of cardiovascular events (3C-MACE, CV death, MI, stroke, HHF) needs to be further investigated.

Although all clinical studies carried out with the DPP4i confirmed their cardiovascular safety, only a neutral effect on the reduction of cardiovascular risk was observed in T2D patients with a history of CVD. A critical question is why no cardioprotective outcomes were observed for DPP4i in clinical trials compared to GLP1-RA although the baseline characteristics of the study populations for GLP1-RA and DPP4i were similar (Table 1). Trials included T2D patients with CVD and eGFR in the range of 71–80 mL/min per 1.73 m^2^, indicating mildly impaired kidney function (EXAMINE, TECOS, Harmony Outcomes, ELIXA). An important aspect might be the difference in the effectively reached GLP1-RA levels and the duration of agonist availability in both therapies. Treatment with DPP4i leads to modest increase in endogenous GLP1 plasma levels (2–3-folds) although the enzyme activity is up to 90% reduced. In contrast, application of a synthetic GLP1-RA results in a remarkably higher plasma concentration of the GLP1-RA (8–10 folds) and a prolonged effect due to the improved half-life of the peptides (Gallwitz, 2019). It is important to note the large variations in plasma stability of the chemically modified GLP1-RA, which could be a reason for the lack of cardiovascular benefit on MACE or cardiovascular death with the short-acting agonist lixisenatide (3 hour plasma half-life) in the ELIXA trial, in contrast to liraglutide (11–15 h plasma half-life) in the LEADER study (Bolli and Owens, 2014). Furthermore, an altered GLP1 receptor (GLP1R) activation by the chemically modified GLP1-RA in comparison to native GLP1 may affect the outcomes. *In vitro* studies demonstrated an increased receptor residence time of lixisenatide, liraglutide, dulaglutide and semaglutide in comparison to the native GLP1 as well as small changes in receptor downstream signaling (Jones et al., 2018). Apart from degradation of GLP1, other factors could be involved in the DPP4i-induced blood glucose-lowering effect. This is indicated by results showing that DPP4i lead to anti-hyperglycaemic effects even in mice lacking GLP1R, and that GLP1R antagonism only results in partial inhibition of DPP4i effect (Aulinger et al., 2014). These findings may hint toward different outcomes with DPP4i and GLP1-RA due to different mechanisms of action. Therefore, DPP4i may be second-choice medication behind GLP1-RA and SGLT2i. However, the excellent safety profile of DPP4i, mostly applied in combination with further medication such as metformin, sulfonylurea or thiazolidinediones (with the advantage of DPP4i to induce less weight gain), makes this class of drugs an important treatment option in T2D, especially in patients with CKD or metformin intolerance (Makrilakis, 2019). DPP4i can be used in patients with severe renal impairment (eGFR < 30 mL/min per 1.73 m^2^), especially, linagliptin and vildagliptin (Russo et al., 2013), when metformin was associated with increased mortality in patients with eGFR < 30 mL/min per 1.73 m^2^.

Although clinical trials (Table 1) and more recent meta-analyses (Bergmark et al., 2019; Han et al., 2019) provide evidence for a cardioprotective effect of metformin on the cardiovascular outcome in T2D patients, the available data are less comprehensive compared to DPP4i, GLP1-RA, and SGLT2i (Harrington et al., 2018). The justifiable advantage of metformin is the long experience with the drug in clinical practice, its proven safety and its beneficial influence on a variety of different risk factors in T2D patients, including the reduction of bodyweight, cholesterol levels and all-cause mortality in addition to blood glucose-lowering (Meaney et al., 2008; Herrington et al., 2018). However, due to the exciting CVOT data for GLP1-RA and SGLT2i, metformin as first-line medical therapy for T2D patients with ASCVD is now under review because the evidence of cardiovascular benefit appears weak (Harrington et al., 2018).

## Mechanisms of Cardioprotection of the Antidiabetic Drugs

**FIGURE 1:**
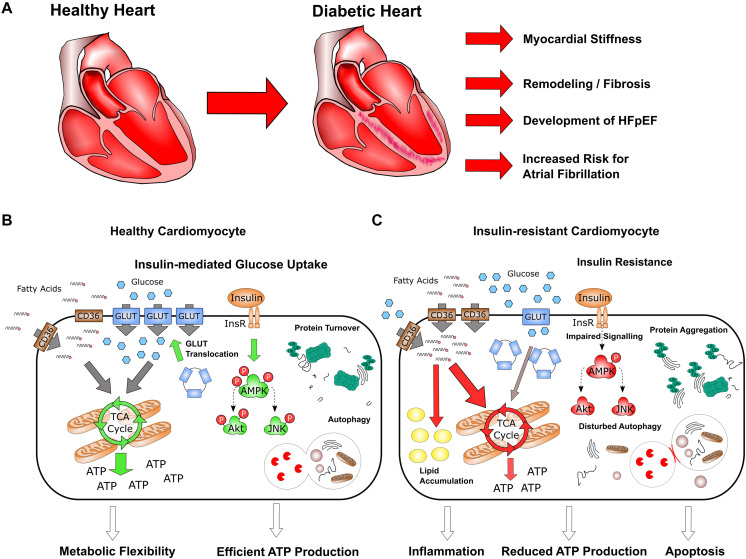
Characteristics of heart failure in diabetes. **(A)** Long-time diabetes is associated with structural remodeling, fibrosis and increased myocardial stiffness in the heart, which lead to the development of HFpEF as well as increased risk for atrial fibrillation. **(B,C)** On the cellular level, insulin-mediated glucose uptake in healthy cardiomyocytes is of key importance for metabolic flexibility and efficient ATP production **(B)**; insulin resistance strongly impairs metabolism and homeostasis in cardiomyocytes, resulting in reduced ATP production and increased inflammation and apoptosis **(C)**.

On a molecular level, insulin resistance causes substantial changes in the energy metabolism in cardiomyocytes, leading to the loss of substrate flexibility and increased fatty acid (FA) oxidation (Garcia-Ropero et al., 2019). This is accompanied with lipid accumulation, impaired mitochondrial membrane potential and reduced ATP production (Figure 1). These processes not only affect cellular energy levels, but also are accompanied with cardiac fibrosis, myocardial stiffness, inflammation, apoptosis, which finally lead to impaired structural organization and the decrease in heart function (Johnson et al., 2016; Hopf et al., 2018; Peng et al., 2019). Over the years, this comprehensive dysregulation manifests clinically as arrhythmia and HFpEF, which may develop into HFrEF.

Clinical data strongly suggest that SGLT2i and GLP1-RA induce cardioprotection through different mechanisms. This becomes evident by the gradual divergence of the event curves for both drug classes, with a direct risk reduction after initiation of treatment with SGLT2i, whereas GLP1-RA show effects at later time points (Cosentino et al., 2020). The fast incidence of cardioprotection observed for SGLT2i was discussed to be induced by volume reduction, an increase of haematopoiesis and erythropoietin or the occurrence of ketone bodies and resulting switch in cardiac energy metabolism (Garcia-Ropero et al., 2019; Santos-Ferreira et al., 2020). These changes were observed directly with the initiation of the treatment, resulted in a reduced cardiac workload, blood pressure as well as reduced ventricular filling pressures and went in line with the direct onset of cardiovascular benefits (Verma et al., 2019). These recently emphasized cardioprotective effects of antidiabetics lead to the investigation of their mechanisms in different animal models *in vivo* including diabetic, MI and ischemia-reperfusion injury (IRI) models and cardiac cell types *in vitro* (Table 3). Whether SGLT2i, GLP1-RA, DPP4i, and metformin, in addition to their blood glucose-lowering mechanisms, act directly on the cells of the heart is still under debate. Animal models for diabetic heart disease include *db/db* mice as well as obesity and diabetes induced by high-fat diet or streptozotocin (STZ)-induced insulin deficiency (Table 3). In cellular models, the diabetes-associated metabolic imbalance and insulin resistance is induced by cultivation under high-glucose conditions (>25 mM) or high FA levels (Table 3). The type of animal model or the culture conditions for the modeling of diabetic cardiomyopathy *in vitro* are highly relevant to the results and conclusions. As an example, Ramirez et al. demonstrated the beneficial effect of sitagliptin in diabetic, non-obese, non-hypertonic Goto-Kakizaki (GK) Wistar rats by improving glucose metabolism and downregulation of FA metabolism (Ramirez et al., 2018). The results from the animal studies could be reproduced *in vitro* in cardiomyocytes treated with high-FA medium, but not under high-glucose conditions. Thus, it may be difficult to directly compare the effects observed with different compounds under different experimental conditions. Another limitation, especially for the work with isolated cells from rat or mice, is the short incubation time of 12–24 h, which raises a question whether the short-time cultivations of the cells are suitable to recapitulate a disease phenotype that is established over the time course of 5–10 years.

**TABLE 3:** Experimental studies to investigate cardioprotective mechanisms of metformin, GLP1-RA, DPP4i, and SGLT2i.

Treatment	Model	Treatment-induced effects	References
**Animal models**			
**Diabetes**			
Metformin, 4 months	STZ-induced diabetic mice	Reduced autophagy, apoptosis, and fibrosis Reduced Inflammation, **AMPK activation**	He et al., 2013; Yang et al., 2019
Metformin, 3 months	Diabetic GK rats	Reduced fibrosis, and arrhythmia	Fu et al., 2018
GLP1-RA – liraglutide, 2 months	STZ-induced, HFD Wistar rats	Improved heart function, reduced fibrosis	Ji et al., 2014
GLP1-RA – liraglutide, 1 week	HFD induced obese, insulin resistant mice	Reduced fibrosis, and inflammation, AMPK activation, activation of RISK pathway (Akt, GSK3β,Erk1/2), increased eNOS expression	Noyan-Ashraf et al., 2013
DPP4i – sitagliptin, 3 months	STZ induced, HFD Wistar rats	Improved cardiac function, reduced fibrosis, lipid accumulation, inflammation, apoptosis, and arrhythmia	Liu et al., 2015
DPP4i – sitagliptin, 5 months	Diabetic GK rats	Improved insulin sensitivity, and diastolic function, increased glucose uptake, AMPK activation	Ramirez et al., 2018
SGLT2i – empagliflozin, 2 weeks	*db*/*db* mice	Increased cardiac ATP production and glucose oxidation, improved cardiac function	Verma et al., 2018
SGLT2i – dapagliflozin	BTBR *ob*/*ob* mice	Improved cardiac function, reduced inflammation, fibrosis, and apoptosis	Ye et al., 2017
**Myocardial infarction/ischemia-reperfusion injury**			
Metformin	C57BL/6 mice	Reduced infarct size, improved cardiac output	Calvert et al., 2008
GLP1-RA – liraglutide, 1 week before MI	C57BL/6 mice	Reduced infarct size, improved cardiac output	Noyan-Ashraf et al., 2009
DPP4i – linagliptin, 1 week before MI	C57BL/6J mice, *db*/*db* mice	Reduced infarct size, inflammation, fibrosis marker, and apoptosis, improved cardiac output	Birnbaum et al., 2019
SGLT2i – dapagliflozin, 4 weeks before IRI	HFD induced pre-diabetic, obese rats	Reduced apoptosis, ROS, arrhythmia susceptibility, improved heart function	Tanajak et al., 2018
SGLT2i – empagliflozin, 6 weeks before IRI	C57BL/6 mice, HFD	Reduced infarct size, STAT3 activation, independent on Akt, eNOS, Erk1/2, GSK3β	Andreadou et al., 2017
**Direct cellular/tissue effects**			
Metformin, 1 mM	H9c2 cells, high glucose condition	Reduced autophagy, apoptosis, and fibrosis	He et al., 2013
Metformin, 1 μM	H9c2 cells, high glucose condition	Increased glucose uptake, reduced FA uptake	Johnson et al., 2016
GLP1-RA – liraglutide, 100 nM	H9c2 cells, high glucose condition	Reduced ROS, and apoptosis, improved autophagy	Yu et al., 2018
GLP1-RA – GLP1, 25 nM	Neonatal rat CMs, high fatty-acid medium	Reduced lipid accumulation, and apoptosis	Ying et al., 2015
GLP1-RA – GLP1, 100 nM	Isolated rat CMs, high glucose medium	Reduced ROS, no effect on glucose uptake or glycolysis	Balteau et al., 2014
DPP4i – sitagliptin	H9c2 cells, high glucose conditions	Improved autophagy	Zhou et al., 2018
DPP4i – linagliptin	Human CMs and fibroblasts	Reduced inflammasome activation	Birnbaum et al., 2019
SGLT2i – empagliflozin, 0.5–1 μM	Isolated human trabeculae from T2D patients	Reduction of diastolic stiffness, improvement of diastolic function	Pabel et al., 2018
SGLT2i – empagliflozin, 1 μM	Isolated CMs of HF patients	Increased glucose uptake	Mustroph et al., 2019
SGLT2i – dapagliflozin, 0.5 μM	Mouse cardiac fibroblasts, lipopolysaccharide stimulation	Reduced inflammation markers, AMPK activation	Ye et al., 2017

*AMPK, adenosine monophosphate-activated protein kinase; CMs, cardiomyocytes; FA, fatty acid; GK, Goto-Kakizaki; HFD, high-fat diet; IRI, ischemia-reperfusion injury; MI, myocardial infarction; ROS, reactive oxygen species; STZ, streptozotocin.*

**FIGURE 2:**
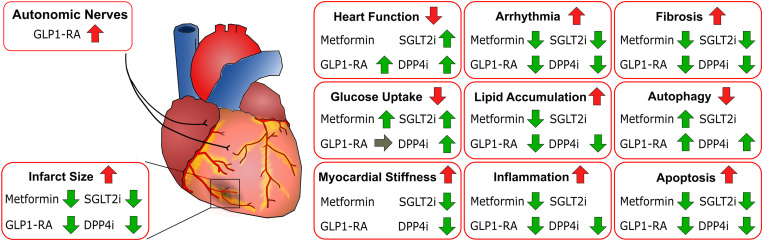
Similar cellular effects of metformin, GLP1-RA, DPP4i, and SGLT2i contributing to cardioprotection. Effects of different drug classes on the heart or cardiac cells in animals and cellular models of diabetes or myocardial infarction. Red arrows indicate the state of the respective aspect under disease conditions. Green arrows indicate the effects observed for treatment.

Many reports for the different classes of antidiabetics highlight the similar cellular effects of metformin, GLP1-RA, DPP4i, and SGLT2i on the heart, which contribute to cardioprotection (Figure 2). Treatment with all compounds was associated with reduced fibrosis, arrhythmia and apoptosis, demonstrating a beneficial effect of all these antidiabetics on the heart (Table 3). Moreover, application of all four classes lead to reduced infarct size and improved heart function in animal models of MI or IRI even in non-diabetic animals. In line with these findings, a reduction in inflammation and lipid accumulation as well as improved autophagy and glucose uptake have been described for metformin, GLP1-RA, SGLT2i, and DPP4i. On a molecular level, compounds from all four classes have been shown to increase the activity of adenosine monophosphate-activated protein kinase (AMPK), one of the central regulators of cellular metabolism (He et al., 2013; Noyan-Ashraf et al., 2013; Balteau et al., 2014; Ye et al., 2017; Ramirez et al., 2018; Yang et al., 2019). The critical role of this activation step in the beneficial effect of metformin, GLP1-RA and SGLT2i was confirmed in studies using specific AMPK inhibitors (Noyan-Ashraf et al., 2013; Ye et al., 2017; Yang et al., 2019), suggesting that the common activation of AMPK represents a key event for cardioprotection of the compounds and that the cardioprotective effects are at least partly independent of blood glucose lowering. From a clinical perspective, the detailed investigation of the underlying mechanisms may be an important rational basis for the specific combination of antidiabetic classes. For example, it is known that metformin stimulates autophagy via activating AMPK and sirtuin-1 (SIRT1) and by this way, may contribute to the cardioprotective effects seen in experimental models of HF (Gundewar et al., 2009). Recent studies revealed that SGLT2i may exert cardioprotective effects by stimulating autophagy (Levine et al., 2015), which may involve the activation of AMPK and SIRT1 (Packer, 2020). The overlap in the mechanism of action between metformin and SGLT2i may be the reason for the reduced cardioprotective effects of SGLT2i in patients with metformin treatment at baseline, when compared to the non-metformin group (Packer, 2020).

Based on the robust cardioprotective effect of GLP1-RA and SGLT2i in clinical studies, understanding their molecular mechanisms is of particular importance. Common effects of GLP1-RA and SGLT2i include the above-mentioned activation of AMPK and reduction of ROS by increased expression of redox-enzymes catalase and superoxide-dismutase SOD2 (Balteau et al., 2014; Andreadou et al., 2017; Mizuno et al., 2018). However, differences between SGLT2i and GLP1-RA are evident in the activation of several downstream signaling pathways. Treatment with SGLT2i led to an activation of STAT3 and reduced levels of IL-6 and inducible NO synthase (iNOS) in the myocardium of mice after ischemia-reperfusion (Andreadou et al., 2017). Furthermore, SGLT2i were shown to improve mitochondrial function, as demonstrated for empagliflozin in mouse MI models (Mizuno et al., 2018) and by dapagliflozin treatment in pre-diabetic rats after ischemia-reperfusion (Tanajak et al., 2018). The *in vitro* studies showed the prevention of TNFα-induced increases in ROS levels and reductions of NO levels by empagliflozin and dapagliflozin, suggesting an important role of endothelial cells for the cardioprotective effect of SGLT2i (Uthman et al., 2019).

The cardioprotective activity of GLP1-RA may involve activation of the reperfusion injury survival kinase (RISK) pathway, which is characterized by activation of the prosurvival kinases phosphatidylinositol-3-OH kinase (PI3K)-Akt and p42/p44 extracellular signal-regulated kinases (Erk1/2) (Rowlands et al., 2018). Liraglutide has been shown to increase phosphorylation of Akt, GSK3β and Erk1/2 in obese, insulin resistant mice (Noyan-Ashraf et al., 2013). In particular, empagliflozin had no effect on the phosphorylation of Akt, GSK3β, and Erk1/2 after ischemia-reperfusion in mice (Andreadou et al., 2017) or Akt- and Erk1/2-activation in *db/db* mice (Habibi et al., 2017). Further mechanisms for the cardioprotection effect of GLP1-RA include beneficial effects on the pathogenesis of arrhythmias and coronary function by increasing cardiac connexin-43 and eNOS levels, as demonstrated by liraglutide in high-fat diet (HFD)-induced insulin-resistant mice (Noyan-Ashraf et al., 2013).

The molecular mechanisms by which cardioprotection can be achieved could vary from drug to drug. Several studies demonstrate that treatment with metformin and DPP4i increases glucose uptake and shifts energy production toward glycolysis by inhibition of key players for FA metabolism as, for example, PDK4, PPARG and CPT1 (Table 3 and Figure 2). Limited data are available for the effect of SGLT2i on cardiac metabolism (Mustroph et al., 2018), and GLP1-RA treatment was not associated with increased glucose uptake in cellular models (Balteau et al., 2014). In addition, beneficial effects on myocardial stiffness have been reported for SGLT2i and DPP4i, accompanied by increasing the phosphorylation of titin which is impaired in diabetic patients (Hamdani et al., 2014; Pabel et al., 2018). Interestingly, metformin was shown to increase phosphorylation of titin (Hopf et al., 2018) but it has not been studied whether metformin reduces cardiac stiffness (Figure 2). These aspects require further investigation to draw general conclusions. Ideally, compounds of the different classes should be examined in parallel in the same model system.

## Direct Cardiac Effects Question the Molecular Target

Experimental studies using isolated cardiac cells or cardiac cell lines provide strong evidence for a direct effect of SGLT2i, GLP1-RA, metformin, and DPP4i on the heart (Table 3). Pabel et al. (2018) demonstrated the direct effect of empagliflozin by immediately reducing the passive stiffness of trabeculae isolated from human end-stage HF patients and improving the diastolic function in mice models directly after injection. These results provide a mechanistic aspect for the early improvement of cardiovascular outcomes with SGLT2i in the clinical studies. A general issue of the *in-vitro* studies represents the concentration of the drug in the experiment, which may strongly exceed the present concentrations *in vivo*.

An ongoing debate about the molecular target of the antidiabetics is not only for newer agents SGLT2i and GLP1-RA, but also for DPP4i or the widely used metformin. Several studies failed to detect SGLT2 in different heart cells (Mustroph et al., 2018; Packer, 2020). An off-target effect of SGLT2i on SGLT1 is also questionable based on the high selectivity of the compounds as well as experiments using phlorizin, a dual SGLT1/2 inhibitor, which could not induce the effects observed with dapagliflozin in isolated cardiac fibroblasts (Ye et al., 2017; Pabel et al., 2018). The observation that SGLT2i reduce cytosolic Na^+^ and Ca^2+^ levels and inhibit the sodium-hydrogen exchanger (NHE) in mouse cardiomyocytes, which are known to be increased in diabetic cardiomyopathy and HF (Baartscheer et al., 2017; Uthman et al., 2018), suggests that the cardioprotective effect of SGLT2i might be due to the direct inhibition of cardiac NHE flux and the reduction of cytosolic Na^+^ and Ca^2+^ levels. Molecular docking studies using a homology model of a bacterial protein structure further suggested a direct binding of SGLT2i to NHE-1 (Uthman et al., 2018). However, this interaction has never been proven using binding assays or site-directed mutagenesis of the putative binding site. Furthermore, reduced bulk cytosolic Ca^2+^ levels by empagliflozin were not observed in human cardiomyocytes (Pabel et al., 2018). Taken together, although a direct effect on the heart has been proven, the molecular target of SGLT2i has not been identified. Off-target effects on SGLT1 and direct binding of SGLT2i to NHE-1 are questionable but the regulation of Na^+^, H^+^, and Ca^2+^ in cardiomyocytes and in specific microdomains may contribute to cardioprotective effects of SGLT2i (Pabel et al., 2018). Of note, the increase in haematocrit and levels of erythropoietin through treatment with SGLT2i were recently discussed to trigger autophagy and reduce reactive oxygen species (ROS) in the heart and thus may represent another indirect effect contributing to cardioprotection (Packer, 2020).

With respect to the target of GLP1-RA, the GLP1R expression is highly relevant. GLP1R is primarily detected in the atria (Ussher et al., 2014). Although detection of GLP1R was shown on mRNA level in ventricles, it is unclear which cardiac cell type expresses functional GLP1R (Ang et al., 2018). Furthermore, GLP1R expression was not found in rat ventricular cardiomyocytes (Wang et al., 2020), but in H9c2 cells (Chang et al., 2018). Ussher et al. provided strong evidence that the cardioprotective effect of GLP1-RA is independent of GLP1R expression in cardiomyocytes (Ussher et al., 2014). Protective effects of liraglutide in a MI model were still present in cardiomyocyte-specific knockout (KO) of GLP1R. Interestingly, cardiomyocyte-specific GLP1R-KO animals showed a lower basal heart rate while liraglutide-induced increase in heart rate was still present (Ussher et al., 2014). In line with these findings, injection of GLP1 increases heart rate and blood pressure in rodents, which has also been reported in some, but not all human trials (Drucker, 2018). This activity of GLP1-RA was linked to direct effects on autonomic nerves (Figure 2). In addition, a recent study in rats indicates an effect of exendin-4 on the ventricular action potential via GLP1R activation on parasympathetic neurons (Ang et al., 2018). In this study, exendin-4 had opposite effects to the stimulation of β-adrenergic receptor, likely through an indirect mechanism mediated by released acetylcholine and nitric oxide, which lead to a reduction of arrhythmia. These findings provide first evidence for the involvement of different processes in the cardioprotective effect of GLP1-RA. However, although the presence of GLP1R in cardiomyocytes seems dispensable for the cardioprotective effect of liraglutide, it is still unclear whether these receptors may be important in other cardiac cell types including endothelial cells, macrophages or fibroblasts, or if other off-target effects may exist.

Further questions arise for the class of DPP4i. The membrane-bound form of the enzyme could not be found in the heart. The broad evidence for cardioprotective effects in pre-clinical studies, especially the effects observed using cell culture models of cardiomyocytes or cardiac fibroblasts, stands in contrast to the lack of efficacy in the CVOTs (Tables 1, 3). Relevant factors to this issue may include species differences, bioavailability of the compounds or the presence of other medication in patients. Importantly, this discrepancy of DPP4i effects may be mechanism-based, because the influence of DPP4 inhibition is, at least partly, indirect and through an increase of the plasma levels of a variety of peptides including GLP1, GIP, neuropeptide Y, peptide YY, gastric inhibitory peptide, or stromal cell-derived factor 1 (SDF-1). An involvement of these indirect downstream effects on the outcome is highlighted by experiments showing that the beneficial effects of saxagliptin in diabetic rats could be prevented by the SDF-1 antagonist plerixafor (Connelly et al., 2016). Moreover, the structural differences of the compounds within the class of DPP4i are relevant for the treatment effect. Saxagliptin, but not sitagliptin, was shown to affect CaMKII/PLB phosphorylation in cardiomyocytes through off-target inhibition of DPP9, which leads to prolonged action potential duration and may trigger arrhythmic events (Koyani et al., 2018). These results indicate that off-target activities of DPP4i on other DPPs may contribute to the different outcome of DPP4i in the clinical trials.

Questions about the molecular target also remain for metformin although it is used in clinical practice since about 25 years. It is widely proven that metformin treatment induces phosphorylation and activation of AMPK (Glossmann and Lutz, 2019). Mechanistically, this has been linked to the ability of metformin to inhibit the activity of mitochondrial complex 1 (MC-1) in the respiratory chain, resulting in an increased AMP/ATP ratio, which triggers AMPK phosphorylation (Glossmann and Lutz, 2019; Soukas et al., 2019). However, the inhibition of MC-1 requires metformin at millimolar concentrations and is therefore critically discussed to fully explain the effects of the drug (Zhang et al., 2016; Soukas et al., 2019). Growing evidence suggests the involvement of other targets including fructose-1,6-bisphosphatase, mechanistic target of rapamycin (mTOR) or mitochondrial glycerol phosphate-dehydrogenase, which are also involved in cellular energy metabolism (Soukas et al., 2019). More recently, an experimental study suggests that the prokineticin (PK) 2/PK receptor (PKR) pathway plays a crucial role in the pathogenesis of diabetic cardiomyopathy and that metformin prevents diabetes-induced glucose and lipid metabolism dysfunction, cardiomyocyte apoptosis, fibrosis, and cardiac insufficiency by stimulating PK2/PKR and the downstream AKT/GSK3β pathway (Yang et al., 2020).

Taken together, although cardioprotective effects were demonstrated for SGLT2i, GLP1-RA, DPP4i, and metformin, the direct targets of the drugs remain elusive and require further investigation. In the future, it is worth to establish novel model systems of diabetic cardiomyopathy, for example, by using induced pluripotent stem cell (iPSC)-derived cardiomyocytes, to investigate the direct effects of the antidiabetics on human cardiomyocytes. The iPSC-based system needs to overcome the challenges of the limited maturity of iPSC-derived cardiomyocytes, but allows long-time cultures to study disease progression in human cells. This may provide sufficient throughput to test different drugs in parallel (Kolanowski et al., 2017). Despite the variety of approaches to model cardiomyopathy hinders the direct comparison of the different drug classes, important information about the cellular pathways involved in cardioprotection could be identified.

## Conclusion

Until recently, T2D and HF were managed independently in clinical practice. The clinical trials clearly confirmed the safety of metformin, GLP1-RA, DPP4i, and SGLT2i (except saxagliptin) for T2D patients at high risk of CVD. The CVOTs demonstrated the cardioprotective effects of GLP1-RA and SGLT2i in T2D patients at high risk of CVD, which strongly encourages clinicians to consider modern T2D therapy in addition to lowering blood glucose levels. Taking into account the baseline characteristics of the patient, especially renal function, atherosclerotic disease or HF, the antidiabetic therapy should be selected in a personalized manner to achieve the best cardiovascular outcome. Moreover, there is an urgent need for further clinical and basic research to decipher and understand the molecular mechanisms of the glucose level-independent cardiovascular benefit observed in the CVOTs.

## Author Contributions

MS, SH, JL, and KG conducted a review of the literature and wrote the first draft of the review. KG contributed to conception and design of the article and finalized the review. All authors read and approved the final manuscript.

## Conflict of Interest

The authors declare that the research was conducted in the absence of any commercial or financial relationships that could be construed as a potential conflict of interest.
